# The Impact of SARS-CoV-2 Infection on the Development of Neurodegeneration in Multiple Sclerosis

**DOI:** 10.3390/ijms22041804

**Published:** 2021-02-11

**Authors:** Angela Dziedzic, Joanna Saluk-Bijak, Elzbieta Miller, Marcin Niemcewicz, Michal Bijak

**Affiliations:** 1 Department of General Biochemistry, Faculty of Biology and Environmental Protection, University of Lodz, Pomorska 141/143, 90-236 Lodz, Poland; joanna.saluk@biol.uni.lodz.pl; 2 Department of Neurological Rehabilitation, Medical University of Lodz, Milionowa 14, 93-113 Lodz, Poland; elzbieta.dorota.miller@umed.lodz.pl; 3 Biohazard Prevention Centre, Faculty of Biology and Environmental Protection, University of Lodz, Pomorska 141/143, 90-236 Lodz, Poland; marcin.niemcewicz@biol.uni.lodz.pl (M.N.); michal.bijak@biol.uni.lodz.pl (M.B.)

**Keywords:** COVID-19, SARS-CoV-2, multiple sclerosis, neurodegeneration, cytokine storm

## Abstract

The novel coronavirus disease 2019 (COVID-19) pandemic, caused by severe acute respiratory syndrome coronavirus 2 (SARS-CoV-2), remains a global challenge. Currently, there is some information on the consequences of COVID-19 infection in multiple sclerosis (MS) patients, as it is a newly discovered coronavirus, but its far-reaching effects on participation in neurodegenerative diseases seem to be significant. Recent cases reports showed that SARS-CoV-2 may be responsible for initiating the demyelination process in people who previously had no symptoms associated with any nervous system disorders. It is presently known that infection of SARS-CoV-2 evokes cytokine storm syndrome, which may be one of the factors leading to the acute cerebrovascular disease. One of the substantial problems is the coexistence of cerebrovascular disease and MS in an individual’s life span. Epidemiological studies showed an enhanced risk of death rate from vascular disabilities in MS patients of approximately 30%. It has been demonstrated that patients with severe SARS-CoV-2 infection usually show increased levels of D-dimer, fibrinogen, C-reactive protein (CRP), and overactivation of blood platelets, which are essential elements of prothrombotic events. In this review, the latest knowledge gathered during an ongoing pandemic of SARS-CoV-2 infection on the neurodegeneration processes in MS is discussed.

## 1. Introduction

Coronavirus disease 2019 (COVID-19) is an infectious disease caused by recently discovered severe acute respiratory syndrome coronavirus 2 (SARS-CoV-2), swiftly leading to a global pandemic. The origin of SARS-CoV-2 infection was first reported in Wuhan, the province of Hubei in China, on 12 December 2019 [1]. According to the World Health Organization (WHO) Situation Reports, as of 17 November 2020 more than 53 million cases across 190 countries and territories have been registered, resulting in more than 1.3 million deaths [2]. Coronaviruses (CoVs) belong to the large *Orthocoronavirinae* subfamily in the *Coronaviridae* family, order *Nidovirales*. Currently, there are four types of CoVs within the *Coronavirinae* subfamily: Alpha-CoV (α-CoV), Beta-CoV (β-CoV), Delta-CoV (δ-CoV), and Gamma-CoV (γ-CoV) [3]. It has been estimated that nearly 2% of the overall human population may be the carriers of CoVs. SARS-CoV-2 belongs to the β-CoV subfamily and is one of the seven currently known CoVs able to cause disease in humans [4]. It is commonly known that α- and β-CoV genuses infect mammals (except pigs) [5], whilst δ- and γ-CoVs primarily (but not exclusively) infect avian species [6]. There are four main CoVs: 229E and NL63 (belonging to α-CoV), as well as OC43 and HKU1 (belonging to β-CoV), which are the most often cause of cold symptoms among people [7]. It is estimated that, 15–30% of common cold cases are due to the CoVs’ infection. Rarely, acute respiratory infections lead to bronchitis and pneumonia and are observed mainly among infants, elders, and immunocompromised people [8].

It was originally believed that SARS-CoV-2 was the cause of myalgia, cough, diarrhea, fatigue, and fever [9]. However, there is a wide spectrum of unusual clinical symptoms of SARS-CoV-2 infection, ranging from asymptomatic or a mild (80% of cases) to severe and critical illness characterized by multi-organ dysfunction and respiratory failure (14% and 5%, respectively) [10]. A wide range of neurological symptoms among those infected with SARS-CoV-2 are observed, some of them are mild, such as a temporary loss of taste and smell, and some are severe, such as encephalopathy [11]. Triggering the inflammatory response is crucial to controlling and combatting the viral infection. In this case, the innate immune system is the first line of human defense. SARS-CoV-2 can productively invade the airways and alveolar epithelial cells, while cell infection derived from hematopoietic lineage, such as monocyte-macrophages and dendritic cells, can also occur but is much more complex [12]. Infection of dendritic cells by SARS-CoV-2 cause moderate upregulation of proinflammatory cytokines, such as tumor necrosis factor-α (TNF-α), interleukin 6 (IL-6), and chemokines (e.g., CC chemokine ligands (CCL)2, CCL3, CCL5, and chemokine (C-X-C motif) ligand (CXCL)10). Additionally, macrophages infected by SARS-CoV-2 show enhanced levels of interferon (IFN)-α and IFN-β as well as other proinflammatory cytokines but with a considerable time delay [13]. Furthermore, in SARS-CoV-2 patients have been found extraordinarily low levels of anti-inflammatory cytokines such as IL-10 [14].

The mechanisms by which SARS-CoV-2 subverts the body’s innate antiviral cytokine responses have yet to be studied, but research on SARS-CoV shows that the variety of viral structural and nonstructural proteins antagonize interferon responses. Likely antagonism occurs at different stages of the interferon signaling pathway, including pattern-recognition receptors (PRRs), which recognize double-stranded viral RNA [15] and (1) prevent PRR signaling through TANK-binding kinase 1(TBK1)/inhibitor of nuclear factor-κB kinase subunit-ε (IKKε), tumor necrosis factor receptor associated factor 3 (TRAF3), and interferon regulatory factor 3 (IRF3) [16,17]; (2) prevent interferon signaling through signal transducer and activator of transcription 1 (STAT1); and (3) promote host mRNA degradation and inhibit host protein translation [18]. It is very likely that at least some of these pathways are conserved in SARS-CoV-2. Activation of immune cells and cytokine storm cause neurovascular inflammations that can have neurological consequences, especially in neurodegenerative diseases such as multiple sclerosis (MS) [19]. MS is characterized by the presence of multifocal zones of inflammation due to local T cells and macrophage infiltrations, as well as oligodendrocyte death being the principal cause of myelin sheath devastation. Neurodegeneration is associated with the abovementioned processes, resulting in the formation of so-called “plaques” in the central nervous system (CNS). Demyelinated neurons and numerous astrocytes are both present in the white and gray matter. The abovementioned lesions, depending on the severity, can lead to partial or total neuronal dysfunction, which is manifested by autonomic and sensorimotor defects, visual disturbances, ataxia, fatigue, or even emotional and psychological problems [20]. 

During the COVID-19 pandemic, an increased risk of a severe course of infection has been observed among patients receiving immunosuppressant or immunomodulatory therapy. MS individuals are in this group. It should be presumed that the higher risk of viral infections may contribute to MS exacerbation and relapses. This study aimed to review the available research describing the common molecular pathways of MS pathomechanisms in the course of SARS-CoV-2 infection.

## 2. Methods and Results

**Figure 1:**
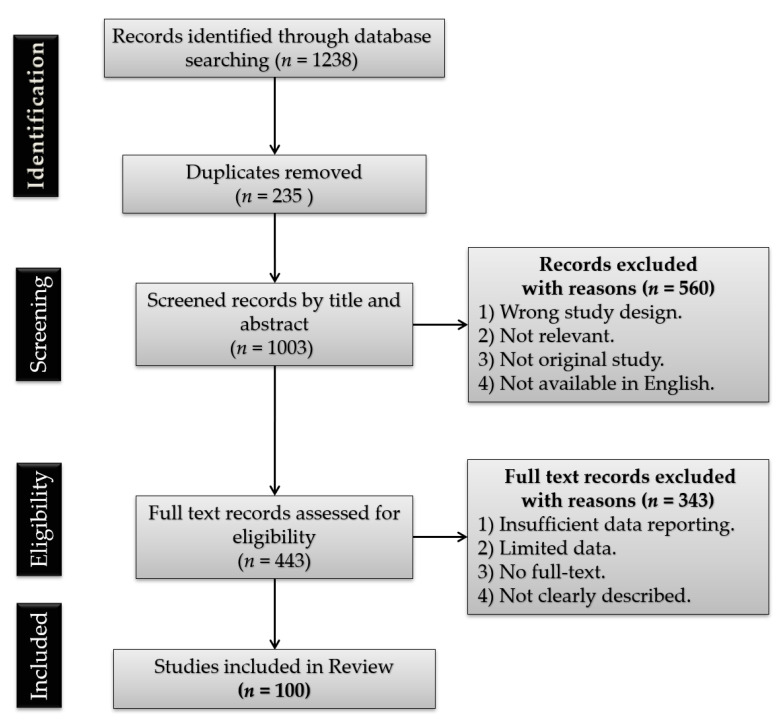
Preferred Reporting Items for Systematic Reviews and Meta-Analyses (PRISMA) flow diagram of the study selection process.

A search for peer-reviewed articles was conducted via the PubMed, Sage Journals, and SCOPUS databases. Google Scholar was also utilized to locate open access articles. The data from the WHO and clinicaltrials.gov websites were also taken into account. The review consists of 163 literature positions, including 100 original research (case reports, clinical trials, cohort studies), 58 reviews (systemic reviews, literature reviews, and meta-analyses), 1 website, and 4 chapters in books. The cited works are in the 1937 to 2021 range of years (most publications come from the year 2020). Identified relevant reviews were hand-searched for additional relevant references. The following search terms were used to locate articles specific to this study: COVID-19, SARS-CoV-2, neurodegeneration, neuroinflammation, multiple sclerosis, cytokine storm, thrombosis, and coagulopathy. Variations of these terms were used to ensure exhaustive search results. After identifying all the keywords, synonyms, and phrases, the Boolean operators “AND” and “OR” were used. The PubMed search was performed using terms and database-appropriate syntax: “SARS” OR “severe acute respiratory syndrome” OR “SARS-CoV-2” OR “coronavirus infections” OR “COVID-19” OR “2019-nCoV” AND “multiple sclerosis” OR “cytokine storm” OR “neuroinflammation” OR “neurological complications” OR “neurodegeneration” OR “coagulopathy” OR “thrombosis”. The preprint server medRxiv for all papers using the search terms “SARS-CoV-2 or coronavirus or COVID-19” was also explored. Furthermore, the search terms relating to “multiple sclerosis” were not useful as the search facilities were not precise. Based on the PRISMA (Preferred Reporting Items for Systematic Reviews and Meta-Analyses) template, a flow chart in order to visualize the studies selection process was created (Figure 1).

## 3. Neuroinflammation and Cytokine Storm in SARS-CoV-2 Infection

The group of CoVs demonstrates a potential neurotropism, which displays a strong affinity to penetrate the CNS, resulting in varying severity clinical features, including encephalopathy, polyneuropathy, and ischemic stroke. It is believed that, the course of COVID-19 disease may have a large neurogenic component due to neurological symptoms observed in people, who have previously not been diagnosed with any type of nervous system disorders [21]. COVID-19 is not only capable of causing pneumonia, but may also lead to impair of other organs, such as kidneys, liver and heart, as well as many organ systems, including circulatory and immune system [22]. Patients ultimately die of multiple-organ failure, acute respiratory distress syndrome (ARDS), cardiovascular incidents, arrhythmias (causing sudden cardiac death), septic shock and renal failure [23]. Dysregulated immune responses along with metabolic dysfunction leading to multiple-organ failure, seem to be the hallmark of the acute state of COVID-19. Studies on the CNS invasion by neurotropic viruses (to which SARS-CoV-2 belongs), and identification of the underlying mechanism leading to neuroinflammation and emerging neurological symptoms have made tremendous strides in recent years. These studies may guide the key areas of investigation in order to explain whether and how SARS-CoV-2 affects the CNS. Brain inflammation is one of the consequences of viral infection caused by herpes simplex, West Nile and Zika resulting in extend-lasting CNS inflammatory processes. The most threatening is the fact that systemic inflammatory response connected with viral infection lead to the blood-brain barrier (BBB) disruption, allowing proinflammatory cytokines get into the CNS and initiates or worsen neuroinflammation leading to encephalitis [24]. Nervous system manifestations including ischemic stroke, cerebral hemorrhage and acute cerebrovascular disease are especially common in severe viral infections [25]. Accumulated evidence suggests that a group of patients with severe COVID-19 demonstrate strong cytokine storm syndrome, which is characterized by increase in the concentration of a huge amount of inflammatory particles, including IL-1β, IL-2, IL-4, IL-6, IL-7, IL-8, IL-10, TNF-α, IFN-γ, granulocyte-macrophage-colony-stimulating factor (GM-CSF), granulocyte colony-stimulating factor (G-CSF), macrophage inflammatory protein 1-α (MIP1-α), MIP1-β, monocyte chemoattractant protein 1 (MCP-1), platelet-derived growth factor (PDGF), vascular endothelial growth factor (VEGF) and inducible protein 10 (IP-10) [26,27,28]. Recently, Chen et al. have demonstrated that, acute SARS-CoV-2 infection is connected with lymphocytopenia (with low CD4+ and CD8+ T-cells levels), increased levels of cytokine IL-2, IL-6, IL-10, TNF-α, and CCL2, and decreased expression of IFN-γ in CD4+ T-cells, similarly to SARS-CoV infection [29]. 

The detailed structural analysis of SARS-CoV-2 demonstrates that, the virus can bind to the angiotensin-converting enzyme 2 (ACE2) receptor, suggesting that it may have parallel pathogenesis to SARS-CoV-1 [30]. It is deliberated that, the key determinant of SARS-CoV specificity is the spike (S) glycoprotein – a protein anchored in the virus envelope responsible for binding to ACE2 receptor. ACE2 receptors are strongly expressed in respiratory system epithelial cells, but also on the neurons and glial cells, making the CNS a potential target for SARS-CoV-2 infection [31]. SARS-CoV-2 uses the receptor ACE2 for entry and the transmembrane serine protease 2 (TMPRSS2) for S protein priming, which is essential for viral spread and its pathogenicity [32]. It is indicated that in mice the protein S from SARS-CoV-2 crosses the BBB by adsorptive transcytosis and the murine ACE2 is involved in brain and lung virus uptake [33].

The prevalence of different inflammatory lung diseases, also those caused by viral infection, is found to be closely sex-dependent in humans [34]. One of the most frequently reported epidemiologic data is focused on differences in sex-related COVID-19 mortality. Significant differences among adult females and adult males (42% vs. 58%) was reported [35]. Several social factors, genetic, immunological, and hormonal differences, as well as lifestyle habits (i.e., smoking and alcohol consumption, chronic diseases etc.), have been considered to play a role in this gender disparity. However, it is strong speculated that the main underlying aspects of these phenomenon is differences in the level of steroid hormones (androgens, estrogen, and progesterone) [36]. Sex-hormone receptors are present on regulatory T-cells [37] and may influence host defense by regulating the ability of immune cells, especially macrophages, to participate in immune responses [38]. The level of testosterone in men remain constant till the age of 30 and afterward it starts to decline with age progression [39]. Testosterone induces the ACE2 expression. Low testosterone level in males has a direct correlation with the disease severity and with worse COVID-19 outcome. Lower levels of testosterone results in the upregulation of ACE2 and TMPRSS2 receptors, facilitating SARS-CoV-1 entry into the alveolar cells, and deregulating a lung-protective pathway [40]. While in female, low levels of androgens may keep low levels TMPRSS2 expression, representing a further protective factor in development of COVID-19 infection. ACE2 expression in females may increase by the X chromosome inactivation escape, due to the fact that the gene encoding ACE2 is expressed on this chromosome [41].

A credible symptom that appears in the early stages of infection, in mildly symptomatic COVID-19 patients, is losing the smell and/or taste [42]. Olfactory and taste disorders (OTDs) in patients with diagnosed COVID-19 are also observed. SARSCoV-2 infection occurs primarily *via* ACE2 receptor, which work as a gateway for the virus’s entry. Enhanced expression of ACE2 in nasopharynx may contribute to superior risk of OTDs [43]. A recently published Italian report found that approx. 20% of hospitalized patients with confirmed COVID-19 had OTDs. Interestingly, OTDs are more frequent in females than males (52.6% vs. 25%; *p* = 0.036, respectively) [44]. Further research revealed olfaction disorder in 12.3% studied COVID-19 patients (8.56% females vs. 3.74% males) [45]. Females would more likely suffer from olfactory disorders in comparison to males (*p* < 0.001). It was documented that in 30.43% SARS-CoV-2-positive women had complete loss of smell and 50.52% had temperate to severe decrease in smell [45]. The same study reported that, in 22.46% cases taste disorders (13.37% females vs. 9.1% males) were registered. The same relationship was observed in the case of olfactory loss, namely females would more likely to suffer from taste disorders compared with males (*p* < 0.001). In 7 cases mild decline, 33 moderate to severe decline and in 2 cases of complete loss of taste were observed [45]. Mean duration of olfactory disorders was 15.57 days, while taste disorders was 11.09 days [45]. This sexual dimorphism can be explained by the fact that the olfactory epithelium is one of the target organs for estrogen. The primary olfactory sensory cells contain specific estrogen metabolic enzymes, which influence on the effect of estrogen on the olfactory epithelium [46]. 

Severe cerebrovascular events were more common among older COVID-19 patients with severe respiratory complications caused by SARS-CoV-2 infection [47]. However, the striking fact is that there is a case of large-vessel strokes in COVID-19 patients younger than 50 years old, that is people not in the stroke risk group [48]. A retrospective study from Wuhan (China) demonstrated that, the incidence of ischemic stroke among hospitalized COVID-19 patients was roughly 5%, including the youngest patient of 55 years old [49]. The single-centered observational study reported that, the hypoxic/ischemic encephalopathy in approximately 20% of 111 patients with a severe course of COVID-19 was observed. An explanation for this phenomenon may be the viral invasion into brain stem structures observed during autopsy examination of infected SARS-CoV humans during 2002–2003 pandemic [50] and may in part cause a neurogenic disorder of respiratory function [51]. In support to this view, it should be mentioned that skeletal muscle injury and delirium observed among COVID-19 patients may influence on respiratory function and causing their impairments [25]. The latest research performed on 214 patients with confirmed SARS-CoV-2 infection demonstrated that nearly 40% of them had neurological manifestations: CNS (24.8%), peripheral nervous system (PNS) (8.9%) and skeletal muscle symptoms (10.7%). Poyiadji et al. lately reported the case of a woman infected of SARS-CoV-2, who after few days with cough, fever, and an altered mental condition, presented severe necrotizing hemorrhagic encephalopathy; the infrequent disorder connected with intracranial cytokine storm and disruption of BBB, interestingly, with no direct viral invasion [52]. A recently published study has reported neurological symptoms in 58 of the 64 observational series patients admitted to the hospital because of ARDS with acute symptoms due to COVID-19 [53]. The neurologic findings such as encephalopathy, confusion and agitation, as well as corticospinal tract signs, were recorded in 8 of the 58 patients (14%) on admission to the ICU and in 39 patients (67%) after withheld of neuromuscular blocker. In two patients, a single acute ischemic stroke was detected after magnetic resonance imaging (MRI). However, obtained data is insufficient to determine, which of these symptoms were due to a cytokine storm and which were characteristic to SARS-CoV-2 infection [53].

## 4. Does Infection of SARS-CoV-2 May Be an Actual Neurodegeneration Agent in MS Patients?

MS is one of the most common neurological disease with an autoimmune basis that affects the CNS and causes serious neurological problems among young adults. MS, due to its complex course, is a very heterogeneous disease, and the newest classification of MS clinical courses proposed in 2014 by Lublin et al. distinguish the two main forms of the disease: relapsing-remitting MS (RRMS; clinically isolated syndrome (CIS) and RRMS patients), and progressive MS (primary progressive MS (PPMS) and secondary progressive MS (SPMS patients)), both either active or inactive [54]. Statistically, clinical outcomes in MS are highly variable. Based on 30 years of observation, Chung et al. demonstrated that after this period 46% have RRMS, 34% have SPMS, and in 20% of cases, MS was the contributing cause of death of these patients [55]. In SPMS, myelin damage gets worse over time, without the chance for remissions, as in the RRMS phase. Compartmentalized inflammation and growing damage to the CNS gradually lead to more and more severe symptoms, which consequently lead to irreversible demyelinating changes [56]. The MS etiology still remains ambiguous; however, it can be deliberated as a multifactorial disease resulting from a genetic predisposition joined with environmental influence [57]. The initial causes of damage in MS are focal immune cell infiltration and cytokine infusion into the CNS white and gray matter tissues. Most studies have demonstrated that T-helper (Th) cells’ (also known as CD4+ T cells) interaction with antigen-presenting cells (APCs) plays a crucial role in the initiation, as well in progression of MS [58]. Association of, among others, pathogen-derived molecules to toll-like receptors (TLRs) present on APCs and production of characteristic cytokines—including interleukins IL-4, IL-12, and IL-23—induce CD4+ T cell differentiation into Th1, Th2, or Th17 phenotypes, inducing “cytokine storm” [59]. It is generally considered that Th1 and Th17 mediators are the main culprits responsible for spreading inflammation in people with MS. Th2 cells secrete the anti-inflammatory cytokines such as IL-4 and IL-13, which reduce pathological inflammation via an increase in repair macrophage (M2) and inhibit the activation of inflammatory macrophages (M1). Th1-derived TNF-α and IFN-γ are two major proinflammatory cytokines essential for innate and adaptive immunity. Furthermore, Th1 cells have the capacity to promote inflammation by inhibiting Th2 differentiation. Th17 induces a great amount of proinflammatory cytokines, including IL-17, IL-21, IL-22, and IL-26, which support and stimulate the ongoing inflammatory process [60]. Many studies have shown that apart from CD4+ T cells, the CD8+ T cells (cytotoxic T cells) may also be found in MS lesions. CD8 + T cells play a crucial role in MS pathogenesis by increasing vascular permeability, destroying glial cells, and triggering the death of oligodendrocytes [61]. An irresistible immune-mediated mechanism for BBB disruption is due to the effect of T cells, particularly the Th17, which are substantial to begin with and propagate neurovascular dysfunction and MS pathogenesis [62]. 

The pathology of MS begins with increased migration of autoreactive lymphocytes through the BBB and its transition from physiological to a pathological state causing brain immune response [63], which consequently leads to the neurodegeneration process. Local failure in the brain leads to the accumulation of CD8+ cells and formation of plaques around the corpus callosum and lateral ventricles in the subcortical white matter and cortex, as well as in the brainstem, spinal cord, and optic nerves [64]. Accumulation of the T and B cells, macrophages, and plasma cells, together with the proinflammatory cytokines, reinforce the immune response via microglial cells [65,66]. Furthermore, B-lymphoid follicles in the meninges begin the humoral immune response that may lead to cortex damage due to intrathecal antibody production [67]. The SARS-CoV-2 infection in some patients may lead to interstitial pneumonia during this process. CD8+ T cell counts may increase up to 80% of total inflammatory cell population, underlying the significance of this cell type in host defense [68]. The higher the numbers of virus-specific CD8+ T cells accumulated in the lungs can increase patient survival. However, the mean duration for olfactory disorders was 15.57 days, while for taste disorders it was 11.09 days in COVID-19 patients and were highly variable and dependent on disease phase. Additionally, it is highly unique that ∼80% of all CD8+ T-cells during primary SARS-CoV-2 infection were antigen specific. This suggests the existence of some molecular mechanism that activates such a high ratio of CD8+ T cells. Rapid growth of the CD8+ T cell population (responsible for directly attacking and killing virus-infected cells) explains the increased inflammatory response and cytokine storm but ultimately leads to consumption of these cells and worse infection prognosis in the SARS-CoV-2 course [69]. Proinflammatory cytokines may leak into systemic circulation causing extrapulmonary manifestations, ARDS, and multiple organ failure [70]. Novel data have highlighted the essential role for hosts’ Th17 inflammation in the pathogenesis of edema and pneumonia caused by COVID-19 [71]. It has been demonstrated that IL-17 stimulates migration [72] and survival of neutrophils [73], which in turn contribute to the pathogenesis of COVID-19-driven ARDS [74]. IL-6 is crucial for the promotion of Th17 cell differentiation and IL-17A secretion. The enhanced IL-6 level in serum was demonstrated in several studies of COVID-19 [75] and was correlated with patient mortality [76]. IL-6 plays an important role in regulating the balance between IL-17-producing Th17 cells and regulatory T-cells (Treg). The excessive participation of IL-6 may be explained by the overactivation of Th17 cells observed in COVID-19 patients, as Xu et al. demonstrated [77]. At early stages of infection, low levels of IL-6 may contribute to uncontrolled viral replication and cause acute lung pathology [78,79]. 

In principle, demyelination can also be seen without autoimmune response, as well as secondary demyelination without preactivated T cells [80]. Alternatively, MS can be considered as a primary degenerative condition that initiates the myelinating unit (oligodendroglia, their processes, and myelin) and results in neuroinflammation (the “inside-out” hypothesis) [81]. Researchers conducting a study on the cuprizone-induced demyelination in an animal model—enabling the study of oligodendrocytosis in the absence (immuno-suppression) and presence (immuno-protection) of the peripheral immune system—postulate that MS primarily originates from a slow, progressive oligodendrocyte degeneration caused by metabolic dysfunction that leads to subsequent reactive gliosis in the absence of adaptive immune cell response [82]. 

Demyelination in mice can be induced experimentally by pathogens such as a murine coronavirus (CoV, mouse hepatitis virus, MHV) and Theiler’s encephalomyelitis virus (TMEV) [83]. Demyelination may result from virus-dependent lysis of oligodendrocytes or be a consequence of virus clearance mediated by the host immune response [84]. TMEV is a single-stranded virus of the *Picornaviridae* family, which is a natural enteric mouse pathogen that behaves as a neurotropic virus and can replicate and persist within the CNS and provoke chronic inflammatory demyelination with parallel histological features to those observed in MS patients [85]. TMEV persistently infects macrophage/microglia lineage cells, oligodendrocytes, and astrocytes during the chronic infection phase [86]. Consequently, the TMEV-infected macrophage cell line induces acute focal demyelination, and these non-specific recruited cells secrete a number of proinflammatory molecules that would potentially destroy myelin sheath [87,88]. Similarly, extensive cellular infiltration dominated by macrophages was found in post-mortem lung tissue collected from SARS-CoV-2 patients [89]. 

It has been demonstrated that the murine neurotropic CoVs JHM (MHV-4) causes demyelination in animals [90]. The CoVs’ capacity to produce demyelination in experimentally infected animals has led to studies examining the possibility of the presence of human CoVs in the MS brain. CoV strains were suggested to cause immunopathogenic events resulting in demyelination of the CNS. A possible link between CoVs and demyelination in several diseases, including MS, has been reported [91]. Furthermore, some strains were even proposed as an experimental model for MS to further study the mechanisms of demyelination after virus infection [92,93]. The inflammatory state observed after SARS-CoV-2 infection is mainly induced by such proinflammatory cytokines as IL-1, IL-6, and TNF-α and may be directly responsible for glial cells activation [94]. An eventual possibility could be the production of antibodies against microglia cells triggered by the SARS-CoV-2 as a para- or post-infective event. The case report described by Zhao et al. concerns Guillain-Barré syndrome, an inflammatory and demyelination disorder of the peripheral nerves, during SARS-CoV-2 infection [95]. 

**Figure 2:**
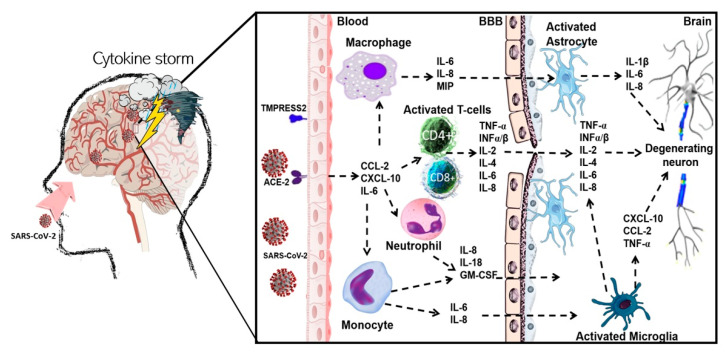
Potential mechanism of severe acute respiratory syndrome coronavirus 2 (SARS-CoV-2)-induced demyelination in human. Coronavirus disease 2019 (COVID-19) is mainly initiated by SARS-CoV-2 through olfactory transmucosal invasion as an entrance into the CNS. The SARS-CoV-2 virus may influence various immune cells, such as macrophages and T cells, as well as being able to interact with endothelial cells via specific receptors ACE2 and TMPRESS2, which work as a gateway for the virus. Such action may be indirectly engaged in BBB damage and demyelination during MS development. SARS-CoV-2 is responsible for recruitment of the massive number of immune cells (especially CD4+ and CD8+ T cells and macrophages) into the CNS, which induce demyelination in MS. Infection of immune cells by SARS-CoV-2 causes severe upregulation of proinflammatory cytokines and chemokines (so called “cytokine storm”), such as IL-1β, IL-2, IL-4, IL-6, IL-8, TNF-α, INF-α/β, MIP1, GM-CSF, CCL5, and CXCL10. Furthermore, macrophages infected by SARS-CoV-2 show increased levels of IFN-α/β as well as other pro-inflammatory cytokines but with a substantial time delay. Abbreviations: angiotensin-converting enzyme 2 (ACE2); blood-brain barrier (BBB); CC chemokine ligands (CCL); central nervous system (CNS); chemokine (C-X-C motif) ligand (CXCL); coronavirus disease 2019 (COVID-19); granulocyte-macrophage-colony-stimulating factor (GM-CSF); interferon (INF); interleukin (IL); macrophage inflammatory protein 1 (MIP1); multiple sclerosis (MS); severe acute respiratory syndrome coronavirus 2 (SARS-CoV-2); transmembrane serine protease 2 (TMPRSS2), tumor necrosis factor α (TNF-α).

To the best of our knowledge, few infectious agents have been connected with MS pathogenesis; however, the immediate implication of a viral agent has never been corroborated. Based on research on SARS-CoV-1, the infection caused by CoVs has been proposed as a potential contributing factor in MS pathogenesis [96]. In preceding epidemics, the presence of human CoV in the brain tissue of patients with MS, as well as in its RNA transcripts, has been demonstrated [97,98,99]. Moreover, the synthesis of human anti-CoV antibodies and human CoV RNA in cerebrospinal fluid (CSF) obtained from MS patients has also been reported [100,101]. CNS demyelination has not been connected with COVID-19 until now; however, the presence of other CoVs was previously associated with MS exacerbations [102], and the participation of autoreactive T cells able to recognize myelin antigens [103]. In the first stage of virus infection in MS, the recruitment of autoreactive Th1/117 cells into the CNS is observed. It suggests that the viral reactivations stimulate the enrolment of pathogenic Th1/17 cells in the CNS. Moreover, autoreactive Th1/17 cells may also induce de novo viral reactivations in a circuit of self-induced inflammation in the host’s CNS. Autoreactive T cells may stimulate viruses to initiate or progress MS, whilst immunopathology could occur later after disease onset [104]. A schematic illustration of the potential mechanism of SARS-CoV-2-induced demyelination in human is represented in Figure 2.

However, MS etiology still needs further investigation, as some association between virus infection and disease initiation and exacerbation was observed [105]. Any microbial and viral infections may lead to body autoimmunity and may in consequence contribute to clinical MS expression, especially in genetically susceptible individuals [106]. There are studies that have implied a potent relationship among viral infections and exacerbation in the MS course [107,108]. Several viruses have been linked with MS pathogenesis, including human herpes virus type 6 [109], Epstein–Barr virus (EBV) [110], and endogenous retrovirus [111]. Oikonen et al. examined the effect of environmental viral infections on the presence of relapses in MS patients. They demonstrated that during influenza A infection there was nearly a 7-fold increase in MS relapses. After EBV infection, the number of relapses in MS was also significantly increased [112]. In other study, Anderson et al. also recorded a correlation between the occurrence of viral upper respiratory and gastrointestinal infections and a significant excess of relapses among 60 MS patients [113]. Patients with comorbidities have a much higher risk for an acute COVID-19 course with acute respiratory failure. The health concerns for MS patients are because of an enhanced risk of infection with SARS-CoV-2 and a more acute form of the disease due to the taking of disease modifying therapies (DMTs) that have immunosuppressive effects in MS [114]. Human CoVs may play a crucial role in MS pathogenesis due to their ability to infect neurons, astrocytes, and glial cells in primary cultures [115,116]. Studies employing MRI have confirmed that the relapses occur in an estimated range of 30–40% of patients after an upper respiratory infection [107,113,117,118,119]. For the first time, Domingues et al. identified the case of a 42-year-old patient with mild respiratory symptoms and neurological manifestations after SARS-CoV-2 infection who had a clinical presentation consistent with CIS. The diagnosis of CIS was based only on the appearance of a clinical attack involving a single anatomical region with no dissemination in space and time. Furthermore, clinical investigation found no oligoclonal bands. This report suggests a likely link between SARS-CoV-2 infection and neurological symptoms of demyelination, even in the lack of characteristic upper respiratory tract infection signs [120]. 

Use of immunosuppressive therapies in MS patients might cause extra risks in the course of COVID-19. However, published case reports noted that taking DMTs was not related with a significantly enhanced risk of hospitalization or fatal outcome [121]. Parrotta et al. demonstrated that the most common COVID-19 symptoms among MS patients taking DMTs were cough and fever; however, 21% of patients had neurologic symptoms return, which occurred coinciding with the SARS-CoV-2 infection. Among the combined number of hospitalized MS patients, 10.5% had severe COVID-19 symptoms. Furthermore, they were in a progressive state of the disease, had comorbidities, as well as were in older age and immobilized. In view of that fact, DMT usage did not correlate with poor COVID-19 outcomes [122]. On the other hand, Dalla Costa et al. estimated the influence of MS treatments on COVID-19 risk individually for each studied drug. In accordance with the results, there was no reliable evidence of worse COVID-19 development in MS patients [123]. Moreover, Novi et al. demonstrated a case report of patient with primary progressive MS who developed COVID-19 and were treated with ocrelizumab. They showed that complete B cell depletion after ocrelizumab led to abated symptoms a few days after hospitalization, with no new symptoms appearing. Interestingly, in spite of the role of immunosuppression, which can increase the risk of disease complications, they postulated that immunosuppressants may potentially treat COVID-19 disease [124]. Additionally, Kloc et al. proposed the protective role of DMTs (used in MS treatment) against ARDS in the course of severe COVID-19. They discovered that clinically accepted MS drugs such as siponimod and fingolimod are strong ACE2 inhibitors [125].

Moore et al. reported the case of a 28-year-old man who had a binocular diplopia after SARS-CoV-2 infection [126]. Initially, the patient reported the typical symptoms of viral disease, such as anosmia, sore throat, cough, myalgias, and headache. A few days later, he noted new symptoms, such as right side oral numbness, vertigo exacerbated by head movements, walking and reading difficulties, and involuntary eye movements causing oscillopsia. These symptoms worsened over the next two days, prompting his admission to an emergency department. He denied any prior episodes of neurological dysfunction, such as visual loss, weakness, ataxia, or paroxysmal sensory or motor symptoms. Nasopharyngeal swab and serum antibody tests obtained upon admission were SARS-CoV-2 positive. Lumbar puncture indicated five unique oligoclonal bands in the CSF that were not present in the serum. MRI demonstrated both contrast-enhancing and non-enhancing white matter lesions in juxtacortical, periventricular, and infratentorial (right paramedian pons) locations. The clinical manifestation of MS was developed during the patient’s recovery from COVID-19-related symptoms. It is the first case reported of patient, who met the revised in 2017 McDonald criteria of dissemination in space and time, required for the diagnosis of MS after SARS-CoV-2 infection [126]. 

There is evidence that suggests relapses associated with infections increase the risk of sustained neurological deficit compared to relapses not associated with infections. Women with MS in the postpartum period are even more vulnerable to relapses and infections. Another case report of SARS-CoV-2 infection in a 40-year-old woman with recurrent RRMS demonstrated that the relapse of MS began right after SARS-CoV-2 infection [127]. Five weeks postpartum, the patient complained of paresthesia and disability in the right limbs, with the onset two weeks before. At admission, hemoglobin and hematocrit were increased 16.5 g/dL (normal range 11.10–14.70 g/dL) and 50.1% (normal range 35–47%), respectively. She had mild lymphopenia and granulocytosis, which worsened during hospitalization, slightly increased CRP level 7.15 mg/L (level less than 5 mg/L is considered negative, while a value more than 10 mg/L is suggestive of positive results), and positive PCR for SARS-CoV-2. She presented a motor deficit and mild ataxia in the right limbs and brisk reflexes; however, her general condition was good, without presenting any of the common cold symptoms. According to the hospital protocol, she was transferred to the infectious diseases hospital designated for the treatment of SARS-CoV-2 positive patients. It was considered an MS relapse with persistent symptoms. The patient began to be administered methylprednisolone 1 g/day for three days. She was discharged after two consecutive negative PCRs. After two weeks, PCR, IgG, and IgM for SARS-CoV-2 were negative, along with the remission of the neurological deficit [127].

One of the case reports proposed that a short course of tocilizumab (600 mg) in acute COVID-19 may be effective, without worsening the preexisting state [128]. A 58-year-old female diagnosed with RRMS in 2007 who was admitted to an emergency department after three days from dry cough and fever appearance, she was tested positive for SARS-CoV-2 by PCR test. She had a very low absolute lymphocyte count (0.33 × 10^9^/L) and remarkably enhanced inflammatory markers, such as IL-6, C-reactive protein (CRP), ferritin, and lactate dehydrogenase (LDH). She had been taking fingolimod since 2011, and after admission to the hospital ward, fingolimod was discontinued owing to concern that immunosuppression may worsen COVID-19. Two days after admission, IL-6 was increased at 23.6 pg/mL (normal < 1.8), and inflammatory markers (CRP, LDH, ferritin, D-dimer) were higher. After three days, the patient developed increasing oxygen needs, and a chest X-ray revealed deteriorating airspace opacities in the lungs. She was transferred to the ICU and took one dose of intravenous tocilizumab (600 mg). Ten days later, she was extubated. Over the following four days, clinical parameters improved and levels of CRP and IL-6 were improved. The patient was discharged four days post-extubation. At the follow-up video visit one week after discharge, the patient continued self-isolation and reported hyposmia and dysgeusia [129]. Fingolimod is a sphingosine-1-phosphate receptor modulator that diminishes the exit of lymphocytes from secondary lymphoid organs into the blood circulation and is related to enhanced risk of virus infection in MS patients [130].

Another case report described a 42-year-old woman with RRMS who reported symptoms of viral infection that gradually worsened over the next few days. Neurologic examination revealed decreased sensation, reduced muscle strength, and positive Babinski sign observed in right foot. These findings were consistent with a new relapse or recrudescence of old MS symptoms (pseudoexacerbation). The SARS-CoV-2 test was positive, and all other medications except hydroxychloroquine were discontinued. The symptoms of cough and dyspnea, together with neurologic symptoms, gradually improved. She was discharged after 13 days of hospital stay and started to take glatiramer acetate in order to avoid any potential MS rebound after the discontinuation of fingolimod. MRI was not retested due to the patient’s hemodynamic instability and a positive SARS-CoV-2 result. It remains unclear whether her neurologic symptoms resulted from new disease activity (relapse) or worsening of preexisting symptoms (pseudorelapse) [131]. In another case report, on 9 March 2020, a 33-year-old woman with RRMS was admitted to the Belgian National Multiple Sclerosis Centrum for neurological assessment and multidisciplinary rehabilitation [132]. The patient had been taking rituximab, an anti-CD20-mediated B cell depleting agent. Seven days after admission, the patient developed the following symptoms: headache, muscle soreness, fever, productive cough, and mild dyspnea. Her test for SARS-CoV-2 via nasal swab was positive. Blood analysis revealed an increased CRP level (35.6 mg/L, normal value < 5.0 mg/L), lymphopenia (0.3 × 10^9^/L, normal range 1.2–3.6 × 10^9^/L), and neutropenia (1.6 × 10^9^/L, normal range 2.5–7.8 × 10^9^/L). The patient completely recovered after symptomatic management over approximately one week. Blood sampling around that time showed a complete restoration of the mentioned above irregularities. After one week following the positive COVID-19 test, the patient demonstrated unchanged levels of CD4+ and CD8+ T lymphocytes, as well as natural killer cells (NKs). CD19 + B cell levels were diminished to 0.3% of the total lymphocyte population (normal range 5–20%). Hypogammaglobulinemia in the IgG subfraction was reported (7.11 g/L, normal range 7.51–15.60 g/L) and D-dimers were enhanced (738 μg/L, normal value < 500 μg/L), according to the previous data in COVID-19 patients. On the basis of the mentioned case, it might be hypothesized that certain forms of immunosuppression provide a protective effect in advance of the cytokine-release storm observed in COVID-19. Hence, certain proinflammatory cytokines, such as IL-2, IL-7, G-CSF, and TNF-α, are increased during viral infection and are connected with unfavorable outcomes in affected MS subjects. That finding demonstrates that SARS-CoV-2 infection does not necessarily translate into a poor prognosis in patients with MS who are receiving B cell-depleting agents, a finding that is compatible with current reports of mild COVID-19 in subjects treated with ocrelizumab. Modulating these pathways might be a favorable approach to this disorder [132].

The pathogenesis of a severe viral infection is closely linked to the development of virus-induced systemic inflammatory response syndrome (SIRS) or SIRS-like disorders and may play a role as an infective trigger of hypoxic neurotoxicity and CNS injuries [133]. Zanin et al. published a case report study of a 54-year-old woman with neurological symptoms including anosmia, dysgeusia, and seizures as consequences of SARS-CoV-2 infection. The presence of multiple demyelinating lesions and alterations of the periventricular white matter with similar lesions (which were found at the bulbo–medullary junction and in both the cervical and dorsal spinal cord) have been confirmed. In addition, autopsy studies revealed SARS-CoV-2 virus particles in the brain [133]. For the first time, Palao et al. discovered CNS demyelination consequences in the form of optic neuritis shortly after COVID-19. In this case, the 29-year-old woman with asthma and rhinoconjunctivitis symptoms was appointed to a physician. The patient had no previously recognized neurological symptoms attributed to SARS-CoV-2 infection, such as anosmia or dysgeusia. During a neurological examination, the patient presented both a visual field defect, as well as a relative afferent pupillary defect in her right eye. In a series of further studies, orbital MRI confirmed a right-sided optic nerve lesion and periventricular demyelinating lesions of the brain. Furthermore, the presence of oligoclonal IgG bands in the CSF was detected [134]. The neurological disorder itself was attributed to viral infection because the autoimmune and serological studies in blood and CSF ruled out other possible etiologies. However, demyelination did not seem to be produced as a result of direct SARS-CoV-2 infection but rather by damage by activation of the microglia and through the generation of inflammatory mediators [134]. 

Yavari et al. described a case report of 24-year-old woman with no family history of neurological problems. During admission to the neurology clinic, the patient was complaining of sore throat, low-grade fever, and myalgia lasting a month [135]. The patient displayed no respiratory symptoms, such as cough and dyspnea. Subsequently, blurred vision and diplopia were added to her symptoms, and the severity of diplopia gradually increased within one day. Such symptoms as fever, sore throat and myalgia continued for the next few days. Afterward, she developed anosmia, corner of the left lip drooping, both upper extremities fingertips paresthesia, left eyelid drooping, and left eyebrow sagging, suggestive of left facial nerve involvement. MRI examination revealed multiple plaques in different brain areas with hypersignal intensity in T2 and fluid-attenuated inversion recovery sequences. According to the patient’s clinical manifestations and the existence of reported plaques, a diagnosis of MS had been clinically suspected. Furthermore, the patient fit the McDonald criteria: one clinical attack and more than two lesions, with objective clinical evidence and dissemination in time observed on MRI (appearance of active plaques in T1 with Gd and plaques in T2). Due to the possible diagnosis of COVID-19 neurological complications, the treatment was started with azithromycin 500 mg daily and naproxen 500 mg daily for one week. After initiating the treatment, fingertips paresthesia and facial nerve paresis improved, without intermittent fever and anosmia [135]. A brief summary of case reports described in this work are included in Table 1 at the end of the next paragraph. 

Current COVID-19 management is supportive and aiming to limit excessive inflammatory response. Secondary hemophagocytic lymphohistiocytosis (sHLH) is an inflammatory syndrome characterized by fatal hypercytokinemia with multiorgan failure. Cardinal features of sHLH include unremitting fever, cytopenias, and hyperferritinemia. A cytokine profile resembling sHLH is associated with COVID-19 severity, characterized by enhanced IL-2, IL-7, INF-γ, MCP-1, MIP-1α, and TNF-α. 

## 5. Coagulopathy and Microvascular Thrombosis in MS Patients after SARS-CoV-2 Infection

MS is typically considered a neurological disease, but it is closely related to vascular damage disorders. As early as in 1937, Tracy Putnam, based on histologic and experimental observations, indicated venular thrombosis as the fundamental pathology found in relation to characteristic MS lesions. The occasional presence of thrombi in severe lesions, with slight plaques surrounding bloated veins and other body organs, was reported in MS patients. Furthermore, Putnam found that most MS patients had a particular defect of the clotting mechanism, proposing that thrombosis was not consequent with vessel wall injury but with blood alterations such as fibrinogen growth [136]. The reports related to vascular disease in MS confirm an increased risk of ischemic events associated with abnormal platelet function and coagulation cascade disorder, especially ischemic stroke, myocardial infarction, and venous thromboembolism [137,138].

The concept of MS being a vascular disease is not new; there are three types of vascular dysfunction in this disorder. Firstly, epidemiological studies established that MS patients are at higher risk of hospital admission due to cardiovascular incidents such as ischemic stroke, myocardial infarction, and heart failure in the first year of the disease’s course, in comparison to the general population [139,140,141]. Secondly, chronic cerebral hypoperfusion (CCH) on itself may contribute to the pathology of MS. Cerebral white matter axons and myelin appear to be especially receptive to chronic hypoxia. It is demonstrated that animals with CCH developed axonal degeneration, focal white matter lesions with apoptosis of oligodendrocytes, myelin decomposition, gliosis [142], and neuronal loss in the hippocampal region [143], which are pathological features of MS. Furthermore, Increasing evidence strongly supports the theory that oxidative stress, largely due to free radicals, induces mitochondrial damage, which arises from chronic brain hypoperfusion [144]. Diminished cerebral perfusion in both gray and white matter of MS patients has already been reported [145,146]. The reduction in perfusion in white and grey matter in MS seems not to be secondary to axonal degeneration but might be a result of diminished astrocyte energy metabolism and decreased axonal activity. Available data suggest that focal MS white matter lesions might have an ischemic origin, and there seems to be a connection among reduced white matter perfusion and cognitive dysfunction [147]. Thirdly and lastly, MS pathology seems to be the consequence of chronic state of injured venous drain from the CNS, the so-called chronic cerebrospinal venous insufficiency (CCVI). It is considered that venous obstruction results in an incorrect flow that promotes inflammation at the BBB and that triggers a disturbance of CNS homeostasis, which leads to demyelination and neurodegeneration [148,149,150]. 

Enhanced risk of cardiovascular diseases in MS patients is correlated with excessive blood platelet activity, leading to increased prothrombotic potential [151,152,153,154]. Moreover, activated platelets due to the interaction with leukocytes begin with increased infiltration of autoreactive T cells, which are responsible for forming new neuroinflammatory lesions in CNS [155,156,157]. Platelets may promote CD4+ T cell differentiation by inhibiting their proliferation, and increase the production of Th1 and support both Tregs and Th17 differentiation, as evidenced by an enhanced level of IL-17 and IL-10 production [158]. During activation, platelets release bioactive factors stored in their granules. As markers of platelet activation in MS, they are considered to be at an elevated level of platelet factor 4 (PF4), CD40L, platelet-activating factor (PAF), and serotonin [159]. Even though platelets do not have a nucleus, they possess the molecular mechanism to synthesize proteins from stored mRNA [160], which may be the perfect tool for implicating the ability to translate proteins from RNA viruses. 

Furthermore, due to their abundance in the circulatory system, blood platelets may be the first blood components that make contact with the SARS-CoV-2 and induce the pathogen response. For example, platelets from influenza-infected patients have been found to contain influenza particles, which like SARS-CoV-2 is a single-stranded RNA (ssRNA) respiratory virus that can infect the alveolar epithelial cells. In human blood platelets, the initial response to ssRNA is mediated predominantly by TLR7 located in the endolysosomes. Due to the influenza infection, one platelet can internalize a high number of viral particles, which colocalize with TLR7 in the lysosomes [161]]. Activation of TLR7 leads to platelet α-granule release in a RAC-alpha serine/threonine-protein kinase (AKT)-dependent manner and accordingly leads to the interaction of platelets with neutrophils via P-selectin and CD40L molecules [162]. Additionally, platelet TLR7 leads to the release of complement C3, which stimulates neutrophils to release their DNA in the process of netosis. Attached neutrophils to the vascular bearing and formats so-called ‘neutrophil extracellular traps’ (NETs). NETs are extremely prothrombotic and, when dysregulated, may induce intravascular coagulation [162]. Thrombin generated from coagulation, in turn, can activate C3 and, consequently, the whole proinflammatory complement cascade [163]. It is demonstrated that COVID-19 serum contains highly specific markers for netosis, which supports intravascular DNA release from neutrophils and their potential applicability to this mechanism [164]. Currently, it is recognized that in patients with severe COVID-19 infection the innate immune response and thrombotic response are closely linked. Noteworthy is the fact that the virus has been detected in general circulation, which proves that SARS-CoV-2 may also enter the CNS via the hematogenous route. The most significant areas of the brain that cause strong expressions of ACE2 in the CNS are perivascular astrocytes [165]. It is shown that perivascular astrocytes are largely eliminated in MS, especially at progressive stages. Kawajiri et al. revealed that ACE2 levels in cerebrospinal fluid from MS patients was significantly diminished compared to healthy individuals [166]. The havoc of astrocytes and low level of ACE2 concentration could theoretically predict ACE2 receptor deficiency, which might reduce the chance of the virus entering into the CNS, and consequently diminish the neurological complications. Paradoxically, this may suggest that neurological complications are less likely to occur in patients with MS in cases who develop COVID-19. However, as with all diseases, it is not possible to simply predict a lower degree of neurological complications in these patients on the basis of one factor, such as a decreased expression of ACE2. An emerging threat related to the COVID-19 pandemic is the tendency of SARS-CoV-2 to cause microvascular, venous, and arterial thrombosis, leading to exacerbation of organ injury. The hyperinflammatory response in patients with severe symptoms of COVID-19 is linked to the development of ARDS and multiorgan failure [167]. As previously mentioned, platelets are essential in maintaining vascular homeostasis and endothelial integrity in the alveolar capillaries, which may contribute to COVID-19 pathophysiology [168]. The ACE2 receptor for SARS-CoV-2 is present in endothelial cells. Besides the tremendous cytokine storm, which has a fundamental impact, some SARS-CoV-2 viral particles can reach and infect the endothelial cells that are strongly ACE2 positive [169]. It is demonstrated that mRNA of SARS-CoV-2 was detected in platelets from patients with COVID-19, despite the lack of mRNA transcripts for ACE2. It suggests that platelets may take up SARS-CoV-2 mRNA independent of the ACE2 receptor [168]. These revelations demonstrate that SARS-CoV-2 infection may be associated with increased platelet activity. Production of proinflammatory cytokines (IL-1β, IL-6, and TNF-α) fundamental in COVID-19 pathology can directly affect platelet function and further contribute to their prothrombotic tendency, even leading to their exhaustion [170]. 

Cardiovascular diseases are frequently observed in patients infected with SARS or Middle East Respiratory Syndrome (MERS) (even up to 30% of cases) [171,172]. SARS-CoV-2 infections tend to downregulate ACE2, which might contribute to myocardial dysfunction, hence affecting the cardiovascular system [173]. Moreover, COVID-19 is associated with myocardial injury, arrhythmias, acute coronary syndrome, and venous thromboembolism [174,175]. Many epidemiological studies confirmed the importance of thrombosis in COVID-19 patients; venous thromboembolism were present in 25–49% of patients with severe viral infection [176,177,178]. Furthermore, statistics showed that the patients with a thrombotic complication have an approximately 5-fold greater mortality [179]. There is no direct link between CoV infection and cardiovascular incidents in the MS course. However, SARS-CoV-2 activity together with the increased prothrombotic potential observed in MS have a potentially overlapping synergistic effect.

Although studies do not implicate SARS-CoV-2 as having a procoagulant effect itself, researchers more likely link COVID-19 coagulopathy to intense inflammation reaction. The coagulation abnormalities associated with COVID-19 mimic other systemic coagulopathies that are regularly observed in severe infections [180]. Violently progressing inflammation, intense activation of the coagulation system, and disturbed balance between pro- and anticoagulant mechanisms can cause generation of disseminated intravascular coagulation (DIC) [181]. DIC as a hypercoagulable state is characterized by extensive activation of the hemostatic system, causing an excessive formation of blood clots in small vessels with simultaneous enormous consumption of blood platelets and coagulation factors, manifested by hemorrhagic sequelae [182]. Many clinical studies have shown a link between SARS-CoV-2 infection and hypercoagulability diagnosis based on abnormalities in coagulation parameters, including elevated fibrinogen and D-dimer levels, elongated prothrombin time (PT), and activated partial thromboplastin time (aPTT); these point the typical clinical picture of so-called “COVID-19 associated coagulopathy” (CAC) in hospitalized patients, even at the early stage of the infection [183]. In advanced COVID-19 infections, elevated levels are often recorded: P-selectin [184], fibrinogen, D-dimer [185,186,187,188], and von Willebrand factor (vWF) [189,190]. Baseline characteristics of 99 patients hospitalized in Wuhan demonstrated that 6% had an enhanced aPTT, 5% increased PT, 36% higher D-dimer level, increased biomarkers of inflammation, including IL-6, erythrocyte sedimentation rate (ERS), and CRP [43]. While such parameters are correlated with a high risk of thrombosis, a rapid drop in fibrinogen level and platelet counts later in a disease course may signal the development of DIC, where bleeding is the major outcome [191]. Tang et al. also confirmed the development of DIC in non-surviving COVID-19 patients who had higher D-dimer levels, fibrinogen degradation products (FDP), and longer PT and APTT values compared to the survivors [192]. Therefore, there is a correlation among elevated acute phase reactants—such as fibrinogen, CRP, and IL-6—which may contribute to COVID-19-associated hypercoagulability. Gao et al. conducted a comparison analysis of the hematological parameters between mild and severe groups of COVID-19 patients that demonstrated crucial differences in IL-6, D-dimer, glucose, thrombin time, fibrinogen, and CRP (*p* < 0.05). Researchers used the receiver operator characteristic (ROC) curve to analyze the early-warning efficiency and the optimal prediction threshold of COVID-19 intensification. They demonstrated that the area under the ROC curve of IL-6 connected with D-dimer was very high, reaching up to 93.3%. It indicated that IL-6 and D-dimer were exactly related to the occurrence of severe COVID-19 in adult patients, and their combined detection had the highest specificity and vulnerability for early prediction of the severity of patients with COVID-19 [193].

## 6. Conclusions

The viral infection-induced inflammatory response may accelerate early and subclinical mechanisms and lead to the development of neurodegenerative processes. ACE2, recognized as a receptor for SARS-CoV-2, is highly expressed in epithelial cells of the respiratory system but also on the neurons and glial cells, making the CNS a potential target for this virus. Based on the knowledge of viral infections so far described, we can support the theory that SARS-CoV-2 infection not only increases the neurological syndrome’s vulnerability but also may promote the development of neurodegenerative disease, especially in individuals that are at high risk. However, additional studies are still needed to better understand the neurotropism of SARS-CoV-2 infection.

## Figures and Tables

**Table 1:** Published case reports of people after SARS-CoV-2 infection and their clinical symptoms and neurological complications.

Ref.	Patient Information	Clinical Symptoms	Neurological Complications	Presumptive Conclusions
[120]	42-year-old woman without neurodegenerative disease diagnosed Comorbidities: -No data	-Coryza and nasal obstruction	-Clinical attack involving a single anatomical region characteristic of clinically isolated syndrome (CIS) type -Paresthesia of the left upper limb	-There is an association between CNS focal symptoms compatible with demyelinating disease after SARS-COV-2 infection, even in the absence of relevant upper respiratory tract infection signs
[126]	28-year-old man without neurodegenerative disease diagnosed Comorbidities: -Glaucoma	-Generalized malaise -Headache	-Binocular diplopia -Anosmia -Right oral numbness -Involuntary eye movements causing oscillopsia -Internuclear ophthalmoplegia -MRI demonstrated both contrast-enhancing and non-enhancing white matter lesions in juxtacortical, periventricular, and infratentorial (right paramedian pons) locations	-The SARS-CoV-2 infection may “unmask” or trigger the MS symptoms even during the acute phase of the infection
[127]	40-year-old woman with relapsing-remitting MS (RRMS) disease duration: 9 years Woman was in the postpartum period. Comorbidities: -No data	-Slightly elevated C-reactive protein (CRP) level -Lack of cold symptoms -Increased hemoglobin and hematocrit levels -Mild lumphopenia and granulocytosis	-Motor deficits -Mild ataxia in the right limbs -Focal neurological deficits -paresthesia	-The patient’s neurological deficits may worsening the previous symptoms in the viral context (relapse mimic) -Women with MS in the postpartum period are even more vulnerable to relapses and infections
[128]	58-year-old woman with RRMS disease duration: 14 years Comorbidities: -Migraine -Diabetes mellitus -Hypertension -Hyperlipidemia -Obesity -Transient ischemic attack	-Dysgeusia -Seizures -Fever -Dry cough -Dyspnea -Multifocal pneumonia -Lymphocytopenia -Airspace opacities in both lungs -ARDS -Cytokine release syndrome (CRS) -Elevated inflammation parameters (IL-6, CRP, ferritin and lactate dehydrogenase (LDH))	-Anosmia -Presence of multiple demyelinating lesions -Alterations of the periventricular white matter with similar lesions (at the bulbo–medullary junction and in both cervical and dorsal spinal cord) -Neuromyelitis optica	-Fingolimod-treated MS patient who developed severe COVID-19 recovered after treatment with tocilizumab
[131]	42-year-old woman with RRMS disease duration: 20 years Comorbidities: -Major depression -Hypothyroidism -Chronic urinary tract infections -Pulmonary embolism	-Fever -Elevated CRP level -Lymphocytopenia -Dry cough -Dyspnea, -Increased respiratory rate -Tachycardia -Ground-glass opacity in lungs	-Worsening of neurologic symptoms -Decreased sensation -Reduced muscle strength	-Appearance of MS relapses following SARS-CoV-2 infection
[135]	24-year-old woman without neurodegenerative disease diagnosed Comorbidities: -no data	-Sore throat -Low-grade fever -Myalgia -No respiratory symptoms -Blurred vision	-Diplopia -Anosmia -Drooping the left lip corner -Paresthesia -Left eyelid drooping -Left eyebrow sagging -active demyelinating plaques in left temporal and right frontal brain areas	-The appearance of demyelinating changes similar to those seen in MS following the SARS-CoV-2 infection
[133]	54-year old woman without neurodegenerative disease diagnosed Comorbidities: -Anterior communicating artery aneurysm (treated surgically 20 years before)	-Headache -Momentary loss of consciousness -Unrest -Interstitial pneumonia -moderate lymphocytopenia -Severe normocapnic hypoxia -seizure	-Anosmia -Ageusia -High Glasgow Coma Scale (GCS) score—12 (normal range 3–15; 12—indicates severe focal sensorimotor deficits -Brain MRI revealed newly diagnosed demyelinating lesions (alterations of the periventricular white matter, hyperintense in T2 weighted image (T2WI), at the bulbo–medullary junction and in both the cervical and dorsal spinal cord)	-SARS-CoV-2-induced delayed immune response -SARS-CoV-2 can induce brain and spine demyelinating lesions -SARS-CoV-2 may lead to a systemic inflammatory response syndrome (SIRS)-like immune disorder
[134]	29-year-old woman without neurodegenerative disease diagnosed Comorbidities: -asthma -Rhinoconjunctivitis	-Papillitis -Bilateral Hoffmann’s sign	-Orbital MRI confirmed a right-sided optic nerve lesion with significant contrast enhancement -Brain MRI showed sparse supratentorial periventricular demyelinating lesions -presence of oligoclonal IgG bands in the cerebrospinal fluid (CSF) -Worsening in both visual field defect and visual acuity -Pyramidal tract dysfunction -Hyperflexia in the lower limbs	-Presence of demyelinating disease in the form of optic neuritis following SARS-CoV-2 infection

## Data Availability

Not applicable.
